# Supplementary material for: Integrated Approaches to Reveal Genes Crucial for Tannin Degradation in Aureobasidium melanogenum T9
Source: Biomolecules. 2019 Sep 2;9(9):439. doi: 10.3390/biom9090439 (PMC6769594; doi:10.3390/biom9090439)
Supplement: Supplementary file 1 [file biomolecules-09-00439-s001.pdf]

Integrated approaches to reveal genes crucial for tannin degradation in *Aureobasidium melanogenum* T9

Lin-lin Zhang [a, #], Jie Li [b, #], Yi-Lin Wang [c], Song Liu [d], Zhi-Peng Wang [e, *], Xin-Jun Yu [f]

[a] College of Chemistry & Environmental Engineering, Shandong University of Science & Technology, Qingdao, 266510, China.
[b] Laboratory for Marine Fisheries and Aquaculture-Qingdao National Laboratory for Marine Science and Technology, Yellow Sea Fisheries Research Institute, Chinese Academy of Fishery Sciences, Qingdao 266071, China.
[c] College of Science, China University of Petroleum, Qingdao, 266580, China.
[d] Development & Reform Bureau, West Coast New Area, Qingdao 266000, China.
[e] Marine Science and Engineering College, Qingdao Agricultural University, Qingdao 266109, China.
[f] Key Laboratory of Bioorganic Synthesis of Zhejiang Province, College of Biotechnology and Bioengineering, Zhejiang University of Technology, Hangzhou, 310014, China.
* Correspondence: spirit87@163.com
[#] The authors contributed equally.

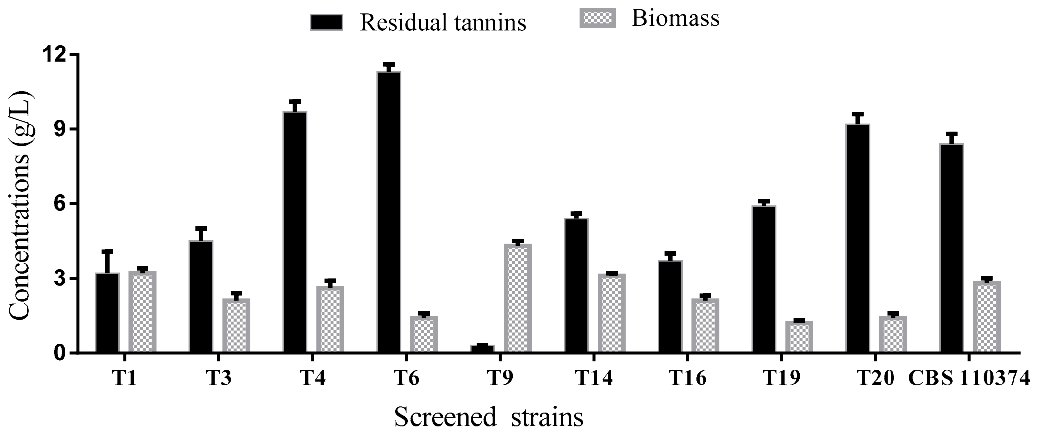

Fig.S1 Tannic acid degradation by different *Aureobasidium* spp. strains

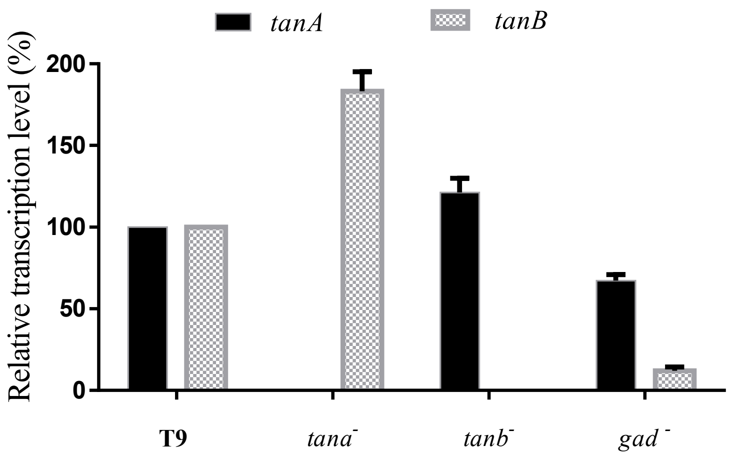

Fig. S2 Transcriptional level analysis of *tanA* and *tanB* genes in different strains
